# Effect of heat treatment on physicochemical, interfacial, and encapsulation properties of pea and soy protein-based emulsions and their spray-dried powders

**DOI:** 10.1016/j.fochx.2025.102791

**Published:** 2025-07-14

**Authors:** Dhananjay Dahatonde, Charu Lata Mahanta, Prashant Kumar, Vikas Mittal, Sanjit Muzumdar, Waseem Khalid, Robert Mugabi, Gulzar Ahmad Nayik, Tawfiq Alsulami

**Affiliations:** a Department of Food Engineering and Technology, School of Engineering, Tezpur University, 784 028, India; b Department of Chemical Engineering, Indian Institute of Technology, Tirupati 517619, India; c Department of Dairy, Dairy Alternatives and Proteins, Kerry group, Kerry Ingredients India Pvt. Ltd., Bengaluru 560103, India; d Research & Development Department, Fresenius Kabi, Gurugram, Haryana 122018, India; e Department of Inorganic, Organic and Biochemistry, Faculty of Chemical Sciences and Technologies, University of Castilla La Mancha, Ciudad Real, 13071, Spain; f Department of Food Technology and Nutrition, Makerere University, Kampala, Uganda; g Marwadi University Research Centre, Department of Microbiology, Marwadi University, Rajkot 360003, Gujarat, India; h Department of Food Science and Nutrition, College of Food and Agricultural Sciences, King Saud University, Riyadh 11451, Saudi Arabia; i University Institute of Food Science and Technology, The University of Lahore, Lahore, Pakistan

**Keywords:** Pea protein concentrate, Soy protein isolate, Plant protein emulsion, High-pressure homogenization, Spray drying, Encapsulation efficiency

## Abstract

Plant proteins are promising alternatives to animal proteins but often show limited emulsion stability after heat treatment. This study evaluated the impact of mild (65 °C) and moderate (85 °C) heating on pea protein concentrate and soy protein isolate emulsions containing sunflower oil, followed by spray-drying. Moderate heating reduced particle size and enhanced zeta potential, protein solubility, and emulsion stability over mild heating. SPI-stabilized emulsions showed higher protein-fat interfacial adsorption and better encapsulation efficiency (94.38 % for S85 vs 82.06 % for P85). Spray-dried powders from moderately heated emulsions exhibited lower sedimentation and retained key physicochemical properties such as smaller particle size, higher zeta potential, and improved encapsulation efficiency. SDS-PAGE revealed differences in protein subunit distribution in the cream phase, indicating altered interfacial protein composition due to heat treatment. These results indicate that moderate heating enhances plant protein functionality, enabling effective encapsulation of oxidation-prone oils into stable, protein-rich powders for food use.

## Highlights

• Moderate heat treatment improved the emulsification properties of pea and soy protein emulsions.
• Pre-heating prior to homogenization enhanced encapsulation efficiency in spray-dried emulsions.
• Soy protein isolate exhibited superior emulsification and encapsulation performance compared to pea protein concentrate.

## 1 Introduction

Proteins are essential macronutrients for human nutrition and are pivotal for maintaining cellular functions and overall physiological health. The global protein ingredients market is expected to reach a valuation of USD 164.3 billion by 2034, rising to a compound annual growth rate (CAGR) of 6.70 % for 2024–2034 (Future Market Insights, 2024). Although animal-derived proteins have long dominated the food industry because of their complete amino acid profiles and high bioavailability, increasing concerns over health risks (e.g., cardiovascular disease, obesity, and cancer), environmental sustainability, and ethical considerations have led to a surge in the demand for plant-based alternatives (Schweiggert-Weisz et al., 2020; Zhong et al., 2021).

Among plant-derived sources, legumes have garnered considerable interest because of their high protein content, functional versatility, and low environmental impact. Legume-based proteins not only offer significant nutritional value but also exhibit desirable functional properties such as solubility, emulsification, gelling, and water/oil-binding capacities (Xu et al., 2023; Zou et al., 2019; Hertzler et al., 2020a). Among these, peas (*Pisum sativum* L*.*) and soybeans (*Glycine* max L.) are among the most widely used legumes in food applications because of their nutritional quality and techno-functional attributes. Pea seeds typically contain 20–25 % protein, 40–50 % carbohydrates, and 10–20 % fiber (Lu et al., 2020). Pea protein is increasingly favored for its digestibility, non-allergenic nature, cost-effectiveness, and balanced amino acid profile (Hertzler et al., 2020). Soybeans, traditionally consumed across East and Southeast Asia, are rich in protein (∼36 %), oil (∼19 %), and carbohydrates (∼35 %), including dietary fiber and essential minerals (Hassan, 2013). Despite having slightly lower sulfur-containing amino acids (e.g., methionine and cysteine), soy protein offers a nutritional profile comparable to that of animal proteins (He et al., 2020). Commercial soy proteins are primarily available in the form of soy protein concentrate (SPC) and soy protein isolate (SPI), with SPI being the most refined and commonly used form in food applications.

Protein-stabilized oil-in-water emulsions are widely utilized to encapsulate bioactive compounds such as polyunsaturated fatty acids (PUFAs), essential oils, flavors, and lipid-soluble vitamins (Rashidinejad & Jafari, 2020; Tupuna-yerovi et al., 2021; Zabot et al., 2022). In such systems, the selection of wall materials is crucial to achieve optimal stability and efficiency. Maltodextrin, frequently employed as a secondary wall material in plant protein-based emulsions, enhances viscosity and water-binding capacity, even though it is not inherently surface-active (Agustinisari et al., 2024; Klinkesorn et al., 2004). Increases viscosity reduces droplet movement and coalescence, thereby improving emulsion stability. It also facilitates homogenization by promoting the formation of smaller droplets (Dokic et al., 2004). Although maltodextrin concentration can influence protein adsorption at the oil-water interface, this effect is minimal at lower concentrations (Yarlina et al., 2024). During spray drying, maltodextrin forms a dense matrix around the oil droplets, enhancing the encapsulation and flowability of the powder. Low dextrose equivalent (DE) maltodextrins are particularly effective in reducing stickiness and moisture absorption, thereby contributing to greater storage stability (Xiao et al., 2022). Among the various carrier oils, sunflower oil is widely favored in emulsion systems because of its high oxidative stability, favorable lipid profile rich in unsaturated fatty acids and tocopherols, and neutral sensory characteristics (Machate et al., 2022; Nakonechna et al., 2024). Encapsulation of sunflower oil enhances oxidative stability of sunflower oil, improves its applicability for various applications especially for use in food, nutraceutical, and pharmaceutical formulations (Le Priol et al., 2022; Sandhya et al., 2023; Srivastava & Mishra, 2021).

Previous research has shown that thermal treatment significantly affects the structural and functional properties of plant proteins. Moderate heating can partially denature proteins, leading to exposure of hydrophobic and sulfhydryl groups, which enhances solubility and interfacial adsorption (Brodkorb et al., 2016; Wang et al., 2012). However, excessive heat may cause aggregation and reduced functionality. Du et al. (2021) and Dybowska and Krupa-Kozak (2020) demonstrated that controlled heat treatments improved emulsion stability and droplet size distribution in protein-based systems. These findings underline the importance of optimizing thermal conditions for plant protein applications.

Following thermal treatment, high-pressure homogenization was employed to prepare the emulsions. This method, which is preferred for industrial-scale applications, produces submicron droplets with narrow size distributions and improved physical stability. Compared to high-speed shear mixing and ultrasonication, high-pressure homogenization provides greater consistency, scalability, and long-term stability (Asua, 2002; Ding & Kan, 2017). Nevertheless, plant protein-based emulsions often demonstrate reduced stability under processing stresses, such as thermal treatment and drying, compared to animal protein-based systems (Karabulut et al., 2024; Kumar et al., 2022; Lima et al., 2023) Thus, investigating the sequential influence of heat-induced structural changes and homogenization on emulsion behavior is crucial for developing robust plant-based encapsulation systems. Subsequently, spray-drying was used to convert the emulsions into powder form, protecting sensitive components such as lipids, flavors, and pigments from oxidation and degradation (Mohammed et al., 2020). Key parameters such as inlet/outlet temperature, feed rate, total solids, and wall material composition significantly influence the powder properties, including moisture content, particle size, and encapsulation efficiency (Mohammed et al., 2020).

Although previous studies have investigated the individual effects of thermal treatment on emulsion stability (Du et al., 2021; Dybowska & Krupa-Kozak, 2020; Lv et al., 2020; S. Zhang et al., 2020), limited research has addressed the combined effects of thermal treatment and high-pressure homogenization on plant proteins such as pea and soy. Understanding how these processing variables influence emulsion characteristics, protein-lipid interactions, sedimentation, and encapsulation performance during spray drying is essential for optimizing plant-based formulations. Therefore, this study aims to explore the influence of mild and moderate heat treatments on the functional behavior of pea and soy protein-based emulsions, processed via high-pressure homogenization. Furthermore, this study evaluates the potential application of resulting spray-dried powders in ready-to-mix plant-based products, including dairy alternatives, functional beverages, and nutraceuticals.

## 2 Materials and methods

### 2.1 Materials

Pea protein concentrate (PPC) was purchased from K. P. Manish Global Ingredients Pvt. Ltd. (Chennai, India), and soy protein isolate (SPI) was obtained from Anshul Life Sciences (Bhiwandi, Maharashtra, India). The color of PPC ranged from creamish to light yellow, while SPI appeared off-white to creamish. The pH values of PPC and SPI in deionized water (prepared using an Elix® Essential water purification system) were 7.35 and 6.45, respectively. Commercial-grade high oleic acid sunflower oil containing 70–90 % monounsaturated fatty acids **(**MUFA) was purchased from AAK Kamani Pvt. Ltd. (Chandivali, Mumbai, India). Low-DE maltodextrin (DE 10–14) was sourced from Roquette India Pvt. Ltd. (Uttarakhand, India). It was used as a filler or secondary wall material to increase the total solids (TS) content and glass transition temperature of liquid emulsions intended for spray drying.

### 2.2 Proximate analysis

Proximate composition analyses of the PPC and SPI powders were conducted according to standard protocols (AOAC, 2019). Moisture content was determined by heating 3.0 g of PPC and SPI samples in a hot air oven at 105 °C until a constant weight was obtained. The protein content (total nitrogen% × 6.25) was determined by the Kjeldahl method, using 0.5 g samples. Fat content was measured using the Soxhlet extraction method with petroleum ether (boiling point range 40–60 °C) as the extractant. Ash content was determined by heating 5.0 g of the samples in a muffle furnace at 550 °C for 6 h. Total carbohydrate content was calculated by difference, using the following formula (1):

(1) $$\text{Total carbohydrate}\left(\%\right)=100-\left(\%\;\text{moisture}+\%\;\text{protein}+\%\;\text{fat}+\%\;\text{ash}\right)$$

### 2.3 Dispersion characteristics of proteins (soluble or non-sedimentable protein)

The dispersion characteristics of the protein samples were determined according to a previously described method by Tan et al. (2019) with some modifications. Protein powder samples (10 g) were dispersed in 150 g of deionized water. The mixtures were adjusted to pH 4.5, 7, and 10.0 with 0.5 M HCl or 0.5 M sodium hydroxide, respectively, and the total weight was then adjusted to 200 g, and the suspensions were stirred for 30 min. Subsequently, the samples were heated to 80 °C in a double-jacketed water bath. After heating, the solutions were centrifuged (Thermo Scientific Heraeus Megafuge 16R) at 5,000 ×*g* for 5 min, and the supernatants were collected and analyzed using the Kjeldahl method. Non-sedimentable protein (NSP) content was calculated using the following formula (2):

(2) $$\mathrm{NSP}\;\left(\%\right)=\frac{\text{Protein in supernatant}}{\text{Total protein in initial sample}}\times 100$$

### 2.4 Emulsion preparation

The emulsion was prepared according to the method described by Du et al. (2021), with slight modifications. A protein-based oil-in-water (O/W) emulsion was prepared to have 30 % total solids in the final emulsion. The formulation included 9 % oil, 4.5 % PPC for the pea protein-based emulsion, or 4.0 % SPI for the soy protein-based emulsion, along with maltodextrin, and 70 % water. To prepare the emulsion, the protein powder (PPC or SPI) was first dispersed in deionized water, followed by pH adjustment to 7.00 ± 0.25 using 0.5 M hydrochloric acid or 0.5 M sodium hydroxide. The suspension was allowed to hydrate for 15 min with continuous stirring. Maltodextrin was then added, and the mixture was allowed to hydrate for 15 min with constant mixing. Finally, the sunflower oil was slowly incorporated and continuously mixing for 15 min. Before homogenization, the mixture was heated to 65 °C for 10 min, followed by heating at 85 °C for 20 min to facilitate emulsion formation. The mixtures were then homogenized using a high-pressure homogenizer (Goma, High-Pressure Homogenizer; Max. capacity 25 LPH) with two passes at zero pressure, followed by three passes at 1 × 10^7^ Pa. The mildly heated protein-based emulsions were labeled as P65 and S65 for PPC and SPI, respectively. In contrast, the moderately heated protein-based emulsions were labeled as P85 and S85 for PPC and SPI, respectively. Images of the protein dispersion suspension and pea and soy protein emulsions are shown in Fig. S1, Fig. S2, and Fig. S3, respectively.

### 2.5 Particle size and zeta potential analysis

The particle size distributions of both fresh and reconstituted spray-dried emulsions were determined using a Malvern Mastersizer 3000. Results were expressed as volume-based average particle sizes: Dv_(10)_, Dv_(50)_, and Dv_(90)_, which represent the particle diameters below which 10 %, 50 %, and 90 % of the total volume of particles are found, respectively (Martins et al., 2020; Tsabet & Fradette, 2015). For emulsion samples, the wet dispersion method was employed using deionized water as the dispersant. Samples were mixed thoroughly and added to the dispersion unit to reach an obscuration level of 5–7 %. A minimum of 10 measurements were recorded, and the average values were reported. Dynamic light scattering (DLS) was performed using a Litesizer DLS 500 instrument (Anton Paar, Graz, Austria) to validate the emulsion particle size results. Emulsions were diluted to a concentration of 0.05 % (*v*/v) at pH 7 and measured at 25 °C. The zeta potential was also determined using a Litesizer DLS 500, following a modified version of the method by Ding and Kan (2017). For this, emulsions were similarly diluted to 0.05 % (v/v) at pH 7, and 1 mL of each sample was transferred into an Omega cuvette for analysis at 25 °C. For spray-dried emulsion powders, approximately 2.0 g of each sample was dispersed in 50 mL of deionized water and analyzed using the same procedures described above to assess the reconstitution behavior and particle size distribution.

### 2.6 Sedimentable protein (SP)

Sedimentable protein was determined according to the method described by Cabezas et al. (2019), which is based on the measurement of non-sedimentable proteins in the emulsion. Freshly prepared emulsions were centrifuged at 12,000 ×*g* for 40 min at 25 °C. After centrifugation, three layers were formed. The uppermost creamy layer and supernatant forming the middle layer were decanted and mixed vigorously (representing the non-sedimentable part of the emulsion). The protein content of this fraction was determined by the Kjeldahl method. The percentage of sedimentable proteins in the emulsion was determined using the following equation (3):

(3) $$\text{Sedimentable Protein}\;\left(\%\right)=\frac{C_{o}-C_{n}}{C_{o}}\times 100$$

Where Co is the total protein content of the emulsion, and Cn is the protein content of the non-sedimentable portion.

### 2.7 Protein content associated with fat

The protein content associated (PAF) with fat in the samples was determined according to the method described by Liang and Tang (2014), with some modifications. One hundred grams of the emulsion was diluted with deionized water in a 1:1 ratio and mixed appropriately. Diluted samples were centrifuged at 12,000 ×*g* for 40 min at 25 °C. After centrifugation, the upper layer of the cream was carefully removed, and the protein content of the cream was analyzed using the Kjeldahl method. PAF was calculated using the following equation (4):

(4) $$\mathrm{PAF}\;\left(\%\right)=\frac{C_{c}}{C_{e}}\times 100$$

Where Cc is the protein content of the cream, and Ce is the protein content of the diluted emulsion.

### 2.8 Sodium dodecyl sulfate-polyacrylamide gel electrophoresis (SDS-PAGE) for proteins associated with fat

SDS-PAGE was performed as described by Nikolic et al. (2012), with slight modifications. The protein fractions associated with fat in the mildly and moderately heated emulsions were determined using SDS-PAGE. For this purpose, emulsions were diluted 1:1 with deionized water and centrifuged at 12,000 ×*g* for 40 min at 25 °C. The cream layer was collected for analysis. To determine the protein fractions associated with fat, native PPC and SPI were analyzed using SDS-PAGE. For this, 100 mg/mL stock solutions of powdered PPC and SPI and emulsion-cream of PPC and SPI emulsions were prepared in deionized water. The samples were then subjected to denaturation at 95 °C for 10 min in the presence of β-mercaptoethanol, which is a reducing agent. After that, samples were loaded into the respective wells of the SDS-PAGE gel (5 % stacking and 12 % resolving gel) and run at 130 V for nearly 80 min. The gels were then stained with Coomassie Brilliant Blue overnight and destained with a destaining solution. Images were captured using a Vilber Lourmat gel documentation system.

### 2.9 Spray drying

The emulsion samples were spray-dried using a Lab Model spray drier (Drying Systems India Pvt. Ltd., Thane, India) equipped with a nozzle-type atomization system with a 1.5 mm diameter and an evaporation rate of 3 L/h. The inlet air temperature was adjusted to 190 °C, compressed air pressure was 2.5 kg/cm^2^, and the outlet temperature was maintained at 115 ± 5 °C by controlling the flow rate. The spray-dried powders were collected in a cyclone collection vessel. Images of the spray-dried emulsion powders are shown in Fig. S4.

### 2.10 Oil encapsulation efficiency (EE)

The EE of the emulsion powder samples was determined according to the method described by Vélez-Erazo et al. (2020), with some modifications. Briefly, 5 g of powder was vortexed for 2 min with 35 mL of petroleum ether, and the mixture was filtered through Whatman filter paper grade 1. The tube was rinsed with 25 mL of petroleum ether and filtered. The solvent was evaporated in a water bath at 90 °C, and then in an oven at 105 °C until a constant weight was achieved. Total oil refers to the mass of oil present in the emulsion powder samples. In contrast, surface oil refers to the mass of oil in the powder particles extracted using petroleum ether. The EE was calculated using the following equation (5):

(5) $$\mathrm{EE}\;\left(\%\right)=\frac{\;\text{Total oil}\%-\text{Surface oil}\%}{\;\text{Total oil}\%}\times 100$$

### 2.11 Statistical analysis

All experiments were performed in triplicate, and the results are expressed as mean ± standard deviation. Statistical significance was validated by Duncan's Multiple Range test (DMRT) at a 95 % confidence level (*p* < 0.05) using IBM SPSS Statistics 23.0 software. Additionally, a two-tailed Student's *t*-test was performed using Microsoft Excel software (version 2013) to compare specific data sets, with significance set at *p* < 0.05.

## 3 Results and discussion

### 3.1 Proximate analysis of pea protein and soy protein powders

The proximate compositions (moisture, protein, lipid, ash, and carbohydrates) of commercially available PPC and SPI are presented in Table 1. The protein contents of PPC and SPI were 77.88 ± 0.04 % and 87.56 ± 0.40 %, respectively. In contrast, PPC exhibited a higher carbohydrate content (10.89 ± 0.10 %) compared to SPI (4.26 ± 0.37 %). While SPI had a significantly higher protein concentration, PPC was richer in carbohydrates. The lipid content in both protein sources was negligible (< 0.5 %). Proximate analysis plays a crucial role in designing the emulsion formulation. It provides essential data on the protein composition, which helps in maintaining constant protein concentration in both pea and soy protein-based emulsions to maintain the same experimental conditions for all experiments.

**Table 1:** Proximate composition of commercially available plant proteins.

Composition (%)	Pea protein concentrate	Soy protein isolate
Moisture	5.71 ± 0.09	4.79 ± 0.03
Protein	77.88 ± 0.04	87.56 ± 0.40
Lipid	0.24 ± 0.01	0.04 ± 0.02
Ash	5.27 ± 0.04	3.38 ± 0.02
Carbohydrate	10.89 ± 0.10	4.26 ± 0.37

### 3.2 Protein dispersion characteristics (soluble or non-sedimentable protein)

Protein dispersion characteristics encompass both sedimentable and non-sedimentable fractions, as well as soluble and insoluble protein components. Among the functional properties, solubility is the most critical, as it directly influences key functionalities such as foaming, emulsification, and gelation (Uluko et al., 2016). Protein solubility is affected by factors such as temperature, pH, ionic strength, and prior processing and extraction methods (Nahar et al., 2017). Soluble proteins have hydrophilic and hydrophobic regions in their structure, contributing to their surface-active behavior. These properties make them effective stabilizers in oil-in-water emulsions. Although complete solubility is not essential, the ability of proteins to interact with both oil and aqueous phases is necessary to form a stable interfacial film.

In this study, the solubility behavior of commercial PPC and SPI was evaluated at different pH values (4.5, 7.0, and 10.0), and under heating at 80 °C with the aim of optimizing their use in emulsion formulations. After centrifugation, visible sedimentation occurred, and the supernatant did not clearly separate but appeared as multiple layers. These layers were classified into sedimentable and non-sedimentable protein fractions, with the sediment-associated protein defined as sedimentable, and the remaining portion as non-sedimentable proteins. The results of these analyses are summarized in Table 2.

**Table 2:** Effect of pH and heat treatment on non-sedimentable protein content of pea and soy dispersions.

Solubility at different pH levels	Non-sedimentable pea protein (%)		Non-sedimentable soy protein (%)	
	Non-heated	Heated (80 °C)	Non-heated	Heated (80 °C)
Water (pH 6.8)	26.04 ± 0.07 ^A^	43.38 ± 0.05 ^A^	19.19 ± 0.46 ^A^	66.14 ± 0.88 ^A^
pH 4.5	5.17 ± 0.11^B^	5.96 ± 0.25^B^	7.28 ± 0.83^B^	8.16 ± 0.45^B^
pH 7	26.12 ± 0.70 ^A^	43.93 ± 0.59 ^A^	22.29 ± 0.82^C^	70.62 ± 0.77^C^
pH 10	37.37 ± 0.76^D^	68.01 ± 0.82^D^	33.65 ± 0.80^D^	94.37 ± 0.81^D^

Values are presented as mean ± standard deviation. Different single letter superscripts (A-D) in the same column indicate significant differences according to Duncan's Multiple Range Test (DMRT) at *p* < 0.05.

Among the pH values of the heated and non-heated samples tested in this study, the lowest protein solubility of PPC and SPI was observed at pH 4.5, as shown in Table 2. The solubility of pea protein in deionized water (26.04 ± 0.07 % for non-heated and 43.38 ± 0.05 % for heated samples) was comparable to its solubility at pH 7 (26.12 ± 0.7 % and 43.93 ± 0.59 %, respectively). For soy protein, the solubility at pH 7 increased from 22.29 ± 0.82 % in the non-heated sample to 70.62 ± 0.77 % in the heated sample. Based on DMRT analysis (*p* < 0.05), no significant difference was observed in the non-sedimentable pea protein content between samples prepared in deionized water and pH 7, irrespective of heat treatment. The maximum protein solubilities of pea protein and soy protein were observed at pH 10. Proteins carry a net positive or negative charge at pH values below or above their isoelectric point, respectively. Above the isoelectric point (at alkaline pH), protein-protein interactions decrease while protein-water interactions increase. This can be attributed to the presence of negatively charged carboxyl groups (COO^−^) on the protein surface, which enhance electrostatic repulsion and facilitate protein solubilization (Li & Xiong, 2021).

In addition, in the Student's *t*-test analysis, it was found that the sedimentation of protein in all the heated samples was significantly lower (p < 0.05) than in the non-heated samples of both pea protein and soy protein. Upon heating, globular protein molecules begin to unfold, exposing hydrophobic groups that lead to protein aggregation. These structural changes contribute to an increase in the viscosity of the protein suspension. This phenomenon may reduce the sedimentation of proteins (Brodkorb et al., 2016; Taylor et al., 2013). Upon exposure to heat, proteins initially denature, unfolding their native structures, and subsequently form gels through interactions such as disulfide bonding, hydrophobic interactions, and ionic bonding (Brodkorb et al., 2016). In Fig. S2, the heat-treated samples exhibit a higher volume of the sedimentable part of proteins, indicating that the proteins had aggregated upon heating with a consequent increase in size and volume. However, due to increased viscosity, these aggregates are flocculated or remain suspended rather than undergoing proper sedimentation. Consequently, the heated protein suspension appears to contain a higher volume of sediment compared to the non-heated one (Nicoud et al., 2015). In addition, it was observed that pea proteins aggregated less than soy proteins as their volume increased. Several factors affect protein aggregation, such as the amino acid composition, ionic strength, pH, and temperature of the suspension. The results showed that a higher amount of non-sedimentable protein remained suspended in heated samples (Chi et al., 2003). The experimental parameters were determined based on the observed protein dispersion characteristics. Emulsions were prepared by heat treatment at the natural pH of the proteins. Although maltodextrin is not surface-active, its presence as a secondary wall material may have influenced protein dispersion and droplet stabilization indirectly by increasing viscosity, thereby reducing coalescence during homogenization and possibly mitigating heat-induced aggregation during thermal processing (Agustinisari et al., 2024; Yarlina et al., 2024).

### 3.3 Particle size and zeta potential analysis

**Fig. 1:**
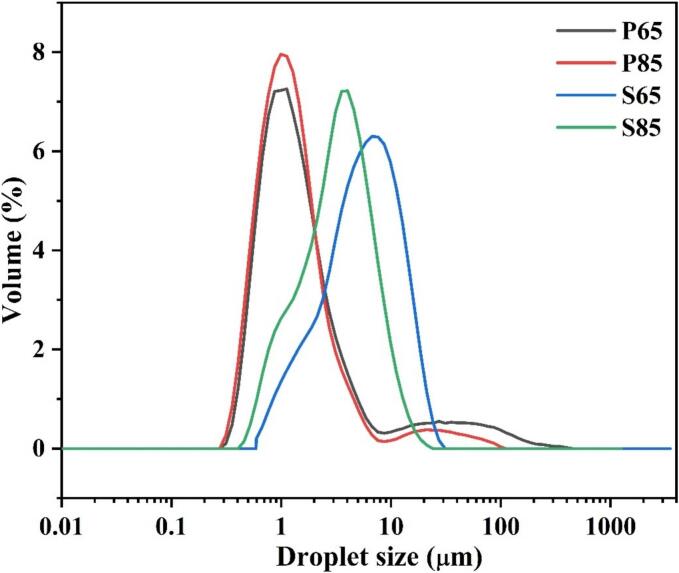
Particle size distribution of mildly and moderately heated pea and soy protein emulsions.

The particle size distribution of all freshly prepared emulsions was measured immediately after emulsion preparation. The particle size is a key parameter that provides valuable information regarding emulsion stability (Faria-Silva et al., 2020; Ursica et al., 2005). The smaller the droplet sizes, typically indicate greater stability (Iyer et al., 2015). The Dv_(10)_, Dv_(50)_, and Dv_(90)_ values for the emulsions stabilized with PPC and SPI are listed in Table 3. The influence of heating at 65 °C and at 85 °C prior to emulsification on the particle size distribution is illustrated in Fig. 1. Dv_(10)_, Dv_(50)_, and Dv_(90)_ represent the droplet diameters (in micrometers) below which 10, 50, or 90 % of the volume of the dispersed phase lies, respectively.

**Table 3:** Particle size distribution of pea and soy protein-based emulsion and spray-dried emulsion powder.

Sample	Particle size distribution of emulsion (μm)			Zeta Potential of Emulsion (mV)	Particle size of spray-dried emulsion powder (μm)		
	Dv_(10)_	Dv_(50)_	Dv_(90)_		Dv_(10)_	Dv_(50)_	Dv_(90)_
P65	0.559 ± 0.036 ^A^	1.199 ± 0.028 ^A^	9.885 ± 0.098 ^A^	28 ± 1.2 ^A^	4.841 ± 0.078 ^A^	18.082 ± 0.18 ^A^	41.321 ± 0.41 ^A^
P85	0.525 ± 0.021 ^A^	1.085 ± 0.04^B^	3.265 ± 0.152^B^	29.7 ± 1.1^B^	3.423 ± 0.1^B^	15.181 ± 0.19^B^	40.243 ± 0.68^B^
S65	1.517 ± 0.017^B^	5.295 ± 0.0.32^C^	12.854 ± 0.113^C^	30 ± 0.8^B^	3.232 ± 0.098^C^	17.322 ± 0.28^C^	49.193 ± 0.73^C^
S85	0.987 ± 0.037^C^	3.139 ± 0.036^D^	7.364 ± 0.09^D^	32.2 ± 1.7^C^	2.912 ± 0.12^D^	14.591 ± 0.11^D^	48.751 ± 0.26^D^

Dv_(10)_, Dv_(50)_, and Dv_(90)_ represent the particle diameters below which 10 %, 50 %, and 90 % of the total sample volume are distributed, respectively.

Data are expressed as mean ± standard deviation. Superscript letters (A-D) within the same column denote statistically significant differences as determined by DMRT (p < 0.05).

The results showed that the Dv_(90)_ values of emulsions prepared with moderately heated PPC (P85) and SPI (S85) were lower, measuring 3.265 ± 0.15 μm and 7.364 ± 0.09 μm, respectively, compared to their mildly heated counterparts, P65 and S65, which exhibited values of 9.885 ± 0.098 μm and 12.854 ± 0.113 μm, respectively. To confirm these results, the particle size was reanalyzed using the DLS method. A similar trend in particle size was observed in Fig. S7. The polydispersity index for P65 and S65 was 35.8 % and 40.6 %, respectively, whereas for P85 and S85, it was 28.2 % and 38 %, respectively (Fig. S7B). The particle sizes of the moderately heated PPC (P85) and SPI (S85) emulsions were smaller than those of the mildly heated emulsions P65 and S65, respectively, which may be attributed to the effect of the heating temperature. The primary protein in pea is legumin (11S), which has a hexameric quaternary structure, and that in soy is glycinin (11S, 7S). Heating at 85 °C of protein-based emulsion prior to homogenization may promote disruption of the original quaternary structure by preventing polypeptide-polypeptide interactions. Researchers have stated that heat treatment of protein-based emulsions prior to homogenization causes disruption of the protein structure and leads to the dissociation of proteins (Keerati-u-rai & Corredig, 2009). The dissociation of proteins may improve the capacity of these proteins to lower the interfacial tension by exposing the hydrophobic regions that are readily adsorbed on the oil droplets (Drusch et al., 2021), and cause a reduction in droplet aggregation in the emulsion. This resulted in the formation of smaller oil droplets in the P85 and S85 emulsions than in the P65 and S65 emulsions.

In zeta potential analysis, it was observed that in mildly heated emulsions P65 and S65, the zeta potential was less than 28 ± 1.2 mV and 30 ± 0.8 mV, respectively, compared to moderately heated emulsions P85 and S85, where the zeta potential was 29.7 ± 1.1 mV and 32 ± 1.7 mV, respectively (Table 3). The difference in zeta potential values between mildly heated (P65 and S65) and moderately heated (P85 and S85) emulsions may be due to structural changes in the proteins caused by heating. Moderate heating can lead to protein unfolding, exposing more charged groups (such as carboxyl and amino groups), which increases the surface charge of emulsion droplets, as reflected by the higher zeta potential values (Lima et al., 2023). It can be hypothesized that the higher zeta potential of moderately heated emulsions favors better stability because of the stronger repulsive forces between the droplets. Industrially, stable plant protein-based emulsions are applicable to plant-based beverages, dairy substitutes, and the creation of meat substitutes that contribute to the overall quality and consumer acceptance of these products.

### 3.4 Sedimentable protein (SP)

After centrifugation at 12,000 ×*g* for 40 min at 25 °C, the emulsions separated into three distinct layers: a creamy top layer, a turbid middle layer (suspension), and a bottom layer containing protein sediment. To determine the amount of sedimentable protein, the protein content of the top two layers (cream and suspension) was measured. The difference between the total protein content and the sum of these layers was considered as sedimentable protein. The results of the protein content analysis are presented in Table 4.

**Table 4:** Percentage of sedimentable protein and protein associated with fat in emulsions.

Sample	Protein content in			Sedimentable protein (%)	Protein content of emulsion cream (%)	Protein content of diluted emulsion (%)	Protein associated with fat (%)
	Emulsion (%) (Co)	Non-sedimentable part of emulsion (%) (Cn)	Sedimentable part of emulsion (%) (Co—Cn)				
P65	3.47 ± 0.11 ^A^	3.24 ± 0.11 ^A^	0.23 ± 0 ^A^	6.63 ± 0.21 ^A^	0.48 ± 0.04 ^A^	1.51 ± 0.08 ^A^	31.47 ± 0.66 ^A^
P85	3.52 ± 0.04 ^A^	3.38 ± 0.06^AB^	0.15 ± 0.02^B^	4.17 ± 0.73^B^	0.76 ± 0.05^B^	1.61 ± 0.09 ^A^	46.89 ± 0.14^B^
S65	3.50 ± 0.08 ^A^	3.32 ± 0.07^AB^	0.18 ± 0.01^C^	5.05 ± 0.23^C^	0.78 ± 0.03^B^	1.77 ± 0.05^B^	43.13 ± 0.53^C^
S85	3.51 ± 0.08 ^A^	3.43 ± 0.10^B^	0.09 ± 0.01^D^	2.47 ± 0.49^D^	0.82 ± 0.03^B^	1.81 ± 0.04^B^	45.11 ± 0.42^D^

Data are expressed as mean ± standard deviation. Different superscript letters (A-D) within a column denote statistically significant differences as determined by DMRT at *p* < 0.05.

The percentage of sedimentable protein was found to be higher in mildly heated emulsions, with P65 and S65 showing 6.63 ± 0.21 % and 5.05 ± 0.23 %, respectively, compared to 4.17 ± 0.73 % for P85 and 2.47 ± 0.49 % for S85 in the moderately heated emulsions. DMRT analysis (*p* < 0.05) confirmed that these differences in sedimentable protein content among the emulsion samples were statistically significant. This reduction in sedimentable protein content can be attributed to the effect of heating. Heat treatment denatures proteins, leading to increased surface hydrophobicity, which enhances their adsorption at the oil-water interface (Taylor et al., 2013). As a result, moderately heated emulsions showed reduced protein sedimentation due to the improved distribution of soluble proteins. These soluble proteins form a thicker interfacial layer around oil droplets, facilitating stronger oil-water interactions and contributing to emulsion stability (Chen et al., 2022).

Moreover, although the total protein content in the emulsion and suspension layer was similar in pea and soy protein systems, there was a significant difference in the protein content in the sediment layers. This highlights the importance of emulsion stability during storage. Once an emulsion is formed, it is important to maintain stability throughout the expected shelf-life, or until the intended application. Stable emulsions offer several functional advantages across various industries, including the controlled release of active ingredients, uniform distribution of ingredients, and improved texture and mouthfeel (Costa et al., 2019; Ishii & Nii, 2014).

### 3.5 Protein content associated with fat

Many researchers have studied the percentage of absorbed protein in emulsions by analyzing the supernatant obtained after centrifugation (Liang & Tang, 2014). However, due to the higher viscosity of the emulsion and the formation of three distinct layers upon centrifugation, the exact amount of protein absorbed onto the fat globules was challenging. To overcome this, the samples were diluted in a 1:1 ratio with deionized water prior to centrifugation, which helped reduce viscosity and improve phase. The protein contents of both the cream layer and the diluted emulsions were analyzed, and the results are presented in Table 4.

The percentage of protein content associated with fat in the mildly heated emulsions (P65 and S65) was 31.47 % and 43.13 %, respectively. In contrast, the moderately heated emulsions (P85 and S85) exhibited higher values of 46.89 % and 45.11 %, respectively. The results showed that heating at 85 °C enhanced protein adsorption onto fat droplets (Fig. S6). This improvement may have occurred because heating at higher temperatures induced the unfolding of proteins that exposed the hydrophobic sites. Fig. S5 indicates the changes in the exposure of hydrophobic sites as measure of ANS (Ammonium 1-anilinonaphthalene-8-sulfonate) fluorescence intensity) thereby altering interfacial behavior. Such structural modifications promote stronger adsorption at the oil-water interface, aligning with previous findings that heat treatment significantly affects the interfacial properties of plant proteins (Wang et al., 2012). DMRT analysis (*p* < 0.05) confirmed significant differences in the protein associated with fat among the different emulsion samples. Enhanced protein adsorption at the oil-water interface contributes to emulsion stability by minimizing destabilization phenomena such as coalescence or flocculation (M. Chen et al., 2022; Zhang et al., 2022). Therefore, moderate heat treatment (85 °C) played a key role in forming more stable emulsions.

### 3.6 Sodium dodecyl sulfate-polyacrylamide gel electrophoresis (SDS-PAGE) of the protein associated with fat

**Fig. 2:**
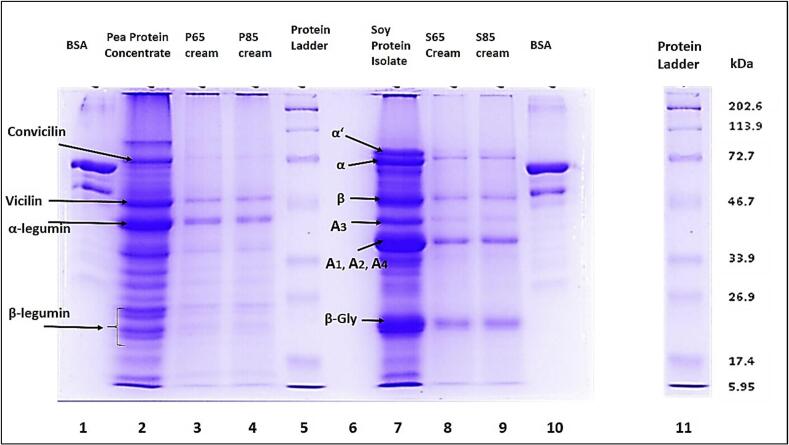
SDS-PAGE of proteins associated with fat under reducing conditions. Lane 1 and 10: BSA; lane 2: pea protein concentrate; lane 3: mildly heated pea protein emulsion cream; lane 4: moderately heated pea protein emulsion cream; lane 5: standard protein; lane 7: soy protein isolate; lane 8: mildly heated soy protein emulsion cream; lane 9: moderately heated soy protein emulsion cream; lane 11: standard protein.

SDS-PAGE was performed to further understand the effect of heating on the interfacial protein composition in oil-in-water emulsions stabilized with pea and soy proteins. Fig. 2 shows the pattern of SDS-PAGE (under reducing conditions) of the cream phase from mildly heated and moderately heated emulsions.

In PPC (lane 2), several bands were observed between ∼21 kDa and ∼ 24 kDa, corresponding to the basic (β-legumin) polypeptide chains (Nikolic et al., 2012). A broad band around 40 kDa represented the acidic (α-legumin) chains. The 7S globulin that is vicilin band was observed at approximately 50 kDa, while convicilin subunits appeared at ∼72 kDa (Boye et al., 2010). In the cream phases of both mildly heated (lane 3) and moderately heated (lane 4) pea protein emulsion, similar bands were present, though less intense, indicating relatively lower protein concentrations or changes in solubility due to heat treatment. For the SPI sample (lane 7), acidic subunits of glycinin (11S), including A_1_, A_2_, and A_4,_ were identified at approximately 36 kDa, while A_3_ was found at ∼42 kDa (Keerati-u-rai et al., 2011; Wang et al., 2012). β-glycinin is a basic subunit of glycinin (11S) with a molecular weight of ∼22 kDa. The bands observed at approximately 80 kDa correspond to the α’ and α-subunits of β-conglycinin (7S). Moreover, the band found at ∼55 kDa corresponded to the β subunit of the β-conglycinin (7S) protein fraction (García et al., 2000; Keerati-u-rai & Corredig, 2009; Liu et al., 2007; Molina et al., 2001). These protein bands were also observed in the cream of mildly (lane 8) and moderately heated (lane 9) soy protein emulsion, however, the bands were faint because of the lower protein concentration. Previous studies have reported that vicilin (7S) in pea protein and β-conglycinin (7S) in soy protein enhance the initial emulsification due to their rapid adsorption at the oil-water interphase, while glycinin (11S) and β-legumin (11S) unfold and adsorb more slowly (Sha & Xiong, 2022; Zhang et al., 2024). In this study, all significant protein fractions may be attributed to the pre-homogenization heating step, which likely caused protein aggregation. Consequently, homogenization distributed all protein fractions uniformly across the oil-water interface, resulting in their presence in the cream layer. However, it should be noted that SDS-PAGE, particularly under reducing conditions using β-mercaptoethanol, only provides information on molecular weight distribution and subunit composition of proteins. It does not directly confirm protein structure or emulsification performance. Therefore, changes observed in the band patterns are interpreted as indicative of altered interfacial protein distribution rather than conclusive evidence of enhanced emulsifying capacity.

### 3.7 Particle size of spray-dried emulsion powders

**Fig. 3:**
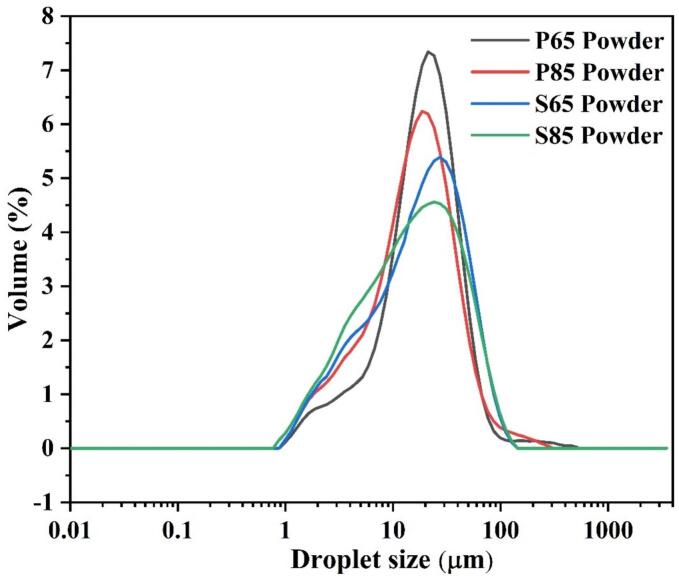
Particle size distribution of mildly and moderately heated pea and soy protein-based emulsion powder.

The particle size of the emulsion powder was measured using the wet dispersion method, and water with a refractive index of 1.330 was used as the dispersing medium. The results are presented in Table 3. The results showed that volume-based particle size distribution (Dv_(90)_) of moderately heated pea and soy protein-based emulsion powders (P85 and S85 powders) had smaller particle size of 40.243 ± 0.68 μm and 48.751 ± 0.26 μm, respectively, compared to mildly heated samples, where P65 and S65 powders exhibited Dv_(90)_ values of 41.321 ± 0.41 μm and 49.193 ± 0.73 μm, respectively (Fig. 3). As discussed in section 3.3, the smaller particle size observed in moderately heated emulsions may be attributed to the heat-induced structural loss and dissociation of proteins, which likely contributed to improved emulsification and subsequent spray-drying behavior.

Previous studies have demonstrated that a smaller particle size enhances EE (Peng et al., 2023), which aligns with our findings (Table 5). Multiple factors can influence the characteristics of spray-dried emulsion powders, including formulation, processing conditions, packaging, and storage. For instance, improper formulation, such as the oil-to-emulsifier or wall material ratios, can lead to inadequate encapsulation (Fang & Bhandari, 2012). This can be avoided by meticulously formulating the emulsion prior to processing. Moreover, processing parameters, such as homogenization and spray-drying, play crucial roles in the characteristics of the powder. Different homogenization techniques yielded different homogenization efficiencies. This affects the particle size and encapsulation of the core material. Additionally, spray-drying conditions, such as inlet air temperature, feed rate, and atomization pressure, affect the powder characteristics. A higher inlet temperature results in larger particles. An increased air-drying temperature establishes the particle structure early and prevents shrinkage during drying, resulting in the formation of larger particles. Some researchers have reported that the amount of surface oil increases with droplet size. Therefore, proper homogenization and optimization of spray-drying conditions are imperative for mitigating these potential disturbances (Piñón-Balderrama et al., 2020; Santos et al., 2018; Vignolles et al., 2009).

**Table 5:** Oil encapsulation efficiency and surface oil content of spray-dried emulsion powders.

Sample	Surface oil content (%)	Total oil (%)	Encapsulation efficiency (%)
P65 powder	6.96 ± 0.12 ^A^	30	76.81 ± 0.39 ^A^
P85 powder	5.35 ± 0.28^B^	30	82.06 ± 0.92^B^
S65 powder	2.44 ± 0.09^C^	30	91.88 ± 0.29^C^
S85 powder	1.69 ± 0.08^D^	30	94.38 ± 0.27^D^

Values are presented as mean ± standard deviation. Different superscript letters (A-D) within the same row indicate significant differences according to DMRT at p < 0.05.

### 3.8 Oil encapsulation efficiency (EE)

EE is an essential parameter in spray-dried emulsion powders, as it indicates the amount of core material encapsulated in the powder matrix (Vélez-Erazo et al., 2020). Accordingly, surface oil represents the unencapsulated or loosely bound oil present on the exterior of powder particles. The presence of free oil negatively influences the physical properties of the spray-dried powders, such as flowability, bulk density, and dispersibility, and accelerates lipid oxidation (Bae & Lee, 2008; Esfahani et al., 2019). In this study, total oil was not directly measured; it was assumed that all the initial oil was retained in the powder due to the non-volatile nature of the oil. Hence, potential losses from deposition on the dryer wall or oil degradation during drying were considered negligible. The surface oil content and encapsulation efficiency of the pea protein-based and soy protein-based emulsion powders are presented in Table 5.

The surface oil content of mildly heated emulsion powders (P65 and S65) was higher, 6.96 ± 0.12 % and 2.44 ± 0.09 %, respectively, compared to their moderately heated counterparts (P85 and S85), which showed lower values of 5.35 ± 0.28 % and 1.69 ± 0.08 %, respectively. Additionally, both soy protein-based powders (S65 and S85) had significantly lower surface oil content than the pea protein-based powders (P65 and P85). These differences may be due to the inherently higher hydrophobicity of soy protein, which contains more hydrophobic amino acid residues (Chen et al., 2019). Enhanced hydrophobicity promotes better coverage of oil droplets, thereby increasing the EE. The elevated surface hydrophobicity of protein may result from the exposure of hydrophobic regions during protein extraction and dehydration processes.

The results showed that heat treatment had a notable influence on the EE of the spray-dried emulsion powders. Statistical analysis (*p* < 0.05) revealed significant differences in EE between mild and moderately heated emulsions. Specifically, the EE of moderately heated powders (P85 and S85) was considerably higher, with values of 82.06 ± 0.92 % and 94.38 ± 0.27 %, respectively, compared to the mildly heated counterparts (P65 and S65), which showed EE values of 76.81 ± 0.39 % and 91.88 ± 0.29 %, respectively. This enhancement in EE may be attributed to improved interfacial stability and stronger protein adsorption at the oil-water interface due to heat-induced protein denaturation. Several factors affect the EE of the spray-dried emulsion powders. These include emulsion formulation, protein hydrophobicity, and processing parameters such as temperature, pressure, and shear during emulsification. In addition, spray drying conditions, such as inlet and outlet air temperature, feed rate, and nozzle size, play crucial roles in determining the final EE (Mohammed et al., 2020). According to Soottitantawat et al. (2005), an increase in emulsion droplet size results in an increase in the amount of surface oil content, likely due to the disruption of droplet stability during atomization or drying, leading to oil migration to the particle surface.

## 4 Conclusion

This study demonstrated the critical role of thermal treatment in modulating the functionality of pea and soy proteins in emulsion-based encapsulation systems. The results showed that moderate heating prior to high-pressure homogenization significantly improved emulsion characteristics, including reduced droplet size, enhanced protein-fat interactions, and increased encapsulation efficiency, particularly for soy protein isolates (SPI). These improvements are attributed to heat-induced protein unfolding, which enhances interfacial activity and promotes stronger protein-oil interactions. Among the two plant proteins studied, soy protein exhibited superior performance across multiple functional parameters, making it a more effective wall material for encapsulating lipophilic compounds in spray-dried systems. Notably, the findings emphasize that a carefully controlled pre-treatment strategy can be used to overcome some of the limitations traditionally associated with plant proteins in emulsion stability and encapsulation applications. This has meaningful implications for the development of clean-label plant-based functional ingredients suitable for use in a wide range of nutraceuticals, dairy alternatives, and fortified beverage powders. In future studies, further investigation of the storage behavior, solubility, oxidative stability, and reconstitution properties of spray-dried powders will be essential for validating their commercial potential. Additionally, expanding this approach to other plant protein sources and bioactive compounds may pave the way for novel encapsulation strategies for sustainable food production. Overall, this study contributes to a growing body of knowledge supporting the design of next-generation plant-based encapsulation materials with improved performance and functionality.

## CRediT authorship contribution statement

**Dhananjay Dahatonde:** Writing – review & editing, Writing – original draft, Software, Project administration, Investigation, Data curation, Conceptualization. **Charu Lata Mahanta:** Writing – review & editing, Supervision, Software, Investigation, Formal analysis, Data curation. **Prashant Kumar:** Writing – review & editing, Resources, Project administration, Methodology, Formal analysis, Data curation, Conceptualization. **Vikas Mittal:** Writing – review & editing, Writing – original draft, Validation, Software, Methodology, Formal analysis, Data curation. **Sanjit Muzumdar:** Writing – review & editing, Software, Resources, Formal analysis, Conceptualization. **Waseem Khalid:** Writing – review & editing, Visualization, Validation, Supervision, Software, Resources, Project administration, Formal analysis. **Robert Mugabi:** Writing – review & editing, Validation, Supervision, Software, Project administration, Methodology, Funding acquisition, Formal analysis, Data curation. **Gulzar Ahmad Nayik:** Writing – review & editing, Visualization, Validation, Supervision, Software, Resources, Methodology, Investigation, Formal analysis. **Tawfiq Alsulami:** Writing – review & editing, Project administration, Methodology, Investigation, Funding acquisition, Formal analysis, Conceptualization.

## Declaration of competing interest

The authors declare that they have no known competing financial interests or personal relationships that could have appeared to influence the work reported in this paper.

## Data Availability

Data will be made available on request.

## Appendix A Supplementary data

Supplementary material Image 1

## References

[bb0005] Agustinisari I., Mulia K., Harimurti N., Nasikin M., Rienoviar H., H., & Manalu, L. P. (2024). The potency of Maillard conjugates containing whey protein as natural emulsifier. International Journal of Food Science.

[bb0010] AOAC (2019).

[bb0015] Asua J.M. (2002). Miniemulsion polymerization. Progress in Polymer Science.

[bb0020] Bae E.K., Lee S.J. (2008). Microencapsulation of avocado oil by spray drying using whey protein and maltodextrin. Journal of Microencapsulation.

[bb0025] Boye J., Zare F., Pletch A. (2010). Pulse proteins : Processing, characterization, functional properties and applications in food and feed. Food Research International.

[bb0030] Brodkorb A., Croguennec T., Bouhallab S., Joseph J.K. (2016). Heat-induced Denaturation, Aggregation and Gelation of whey proteins. Advanced Dairy Chemistry: Volume 1B: Proteins: Applied Aspects: Fourth Edition.

[bb0035] Cabezas D.M., Pascual G.N., Wagner J.R., Palazolo G.G. (2019). Nanoparticles assembled from mixtures of whey protein isolate and soluble soybean polysaccharides. Structure, interfacial behavior and application on emulsions subjected to freeze-thawing. Food Hydrocolloids.

[bb0040] Chen M., Xu F., Nsor-Atindana J., Chen X., Liu F., Wu J. (2022). High protein and high oil emulsions: Phase diagram, stability and interfacial adsorption. LWT.

[bb0045] Chen W., Liang G., Li X., He Z., Zen M., Gao D., Chen J. (2019). Impact of soy proteins, hydrolysates and monoglycerides at the oil/water interface in emulsions on interfacial properties and emulsion stability. Colloids and Surfaces B: Biointerfaces.

[bb0050] Chi E.Y., Krishnan S., Randolph T.W., Carpenter J.F. (2003).

[bb0055] Costa C., Medronho B., Filipe A., Mira I., Lindman B., Edlund H. (2019). Polymers.

[bb0060] Ding Y., Kan J. (2017). Optimization and characterization of high pressure homogenization produced chemically modified starch nanoparticles. Journal of Food Science and Technology.

[bb0065] Dokic P., Dokic-Baucal L., Sovilj V., Katona J. (2004). Influence of maltodextrin dextrose equivalent value on rheological and dispersion properties of sunflower oil in water emulsions. Acta Periodica Technologica.

[bb0070] Drusch S., Klost M., Kieserling H. (2021). Current knowledge on the interfacial behaviour limits our understanding of plant protein functionality in emulsions. Current Opinion in Colloid & Interface Science.

[bb0075] Du Q., Tang J., Xu M., Lyu F., Zhang J., Qiu Y., Liu J., Ding Y. (2021). Whey protein and maltodextrin-stabilized oil-in-water emulsions: Effects of dextrose equivalent. Food Chemistry.

[bb0080] Dybowska B.E., Krupa-Kozak U. (2020). Stability of oil-in-water emulsions as influenced by thermal treatment of whey protein dispersions or emulsions. International Journal of Dairy Technology.

[bb0085] Esfahani R., Jafari S.M., Jafarpour A., Dehnad D. (2019). Loading of fish oil into nanocarriers prepared through gelatin-gum Arabic complexation. Food Hydrocolloids.

[bb0090] Fang Z., Bhandari B. (2012). Encapsulation technologies and delivery Systems for Food Ingredients and Nutraceuticals.

[bb0095] Faria-Silva A.C., Costa A.M., Ascenso A., Ribeiro H.M., Marto J., Gonçalves L.M. (2020). Nanocosmetics.

[bb0100] Future Market Insights (2024). Protein Ingredient Market Growth & Forecast 2024-2034. https://www.futuremarketinsights.com/reports/protein-ingredient-market.

[bb0105] García M.C., Amigo L., Torre M., Marina M.L., Molina E. (2000). Use of phastgel sodium dodecyl sulphate polyacrylamide gel electrophoresis for rapid characterization of soybean proteins in commercial soybean products. Journal of Liquid Chromatography and Related Technologies.

[bb0110] Hassan S.M., El-Shemy H.A. (2013). Soybean-Bio-Active Compounds.

[bb0115] He J., Evans N.M., Liu H., Shao S. (2020). A review of research on plant-based meat alternatives: Driving forces, history, manufacturing, and consumer attitudes. Comprehensive Reviews in Food Science and Food Safety.

[bb0120] Hertzler S.R., Lieblein-Boff J.C., Weiler M., Allgeier C. (2020). Plant proteins: Assessing their nutritional quality and effects on health and physical function. Nutrients.

[bb0125] Ishii F., Nii T. (2014). Colloid and Interface science in pharmaceutical Research and Development.

[bb0130] Iyer V., Cayatte C., Guzman B., Schneider-Ohrum K., Matuszak R., Snell A. (2015). Impact of formulation and particle size on stability and immunogenicity of oil-in-water emulsion adjuvants. Human Vaccines and Immunotherapeutics.

[bb0135] Karabulut G., Goksen G., Khaneghah A.M. (2024). Plant-based protein modification strategies towards challenges. Journal of Agriculture and Food Research.

[bb0140] Keerati-u-rai M., Corredig M. (2009). Food hydrocolloids heat-induced changes in oil-in-water emulsions stabilized with soy protein isolate. Food Hydrocolloids.

[bb0145] Keerati-u-rai M., Wang Z., Correding M. (2011).

[bb0150] Klinkesorn U., Sophanodora P., Chinachoti P., McClements D.J. (2004). Stability and rheology of corn oil-in-water emulsions containing maltodextrin. Food Research International.

[bb0155] Kumar M., Tomar M., Punia S., Dhakane-Lad J., Dhumal S., Changan S., Kennedy J.F. (2022). Plant-based proteins and their multifaceted industrial applications. LWT.

[bb0160] Le Priol L., Gmur J., Dagmey A., Morandat S., Kirat K.E.L., Saleh K., Nesterenko A. (2022). Oxidative stability of encapsulated sunflower oil: Effect of protein-polysaccharide mixtures and long-term storage. Journal of Food Measurement and Characterization.

[bb0165] Li R., Xiong Y.L. (2021). Sensitivity of oat protein solubility to changing ionic strength and pH. Journal of Food Science.

[bb0170] Liang H., Tang C. (2014). LWT-Food science and technology pea protein exhibits a novel Pickering stabilization for oil-in-water. LWT- Food Science and Technology.

[bb0175] Lima R.R., Stephani R., Perrone Í.T., de Carvalho A.F. (2023). Plant-based proteins: A review of factors modifying the protein structure and affecting emulsifying properties. Food Chemistry Advances.

[bb0180] Liu S., Zhou R., Tian S., Junyi G. (2007).

[bb0185] Lu Z.X., He J.F., Zhang Y.C., Bing D.J. (2020). Composition, physicochemical properties of pea protein and its application in functional foods. Critical Reviews in Food Science and Nutrition.

[bb0190] Lv P., Wang D., Chen Y., Zhu S., Zhang J., Mao L. (2020). Pickering emulsion gels stabilized by novel complex particles of high-pressure-induced WPI gel and chitosan: Fabrication, characterization and encapsulation. Food Hydrocolloids.

[bb0195] Machate D.J., Melo E.S.P., S. O., Bogo D., Michels F.S., Pott A., C. A., Freitas, K. de C., Hiane, P. A., Caires, A. R. L., Vilela, M. L. B., Oliveira, R. J., & Nascimento, V. A. do. (2022). Oxidative stability and elemental analysis of sunflower (Helianthus annuus) edible oil produced in Brazil using a domestic extraction machine. Frontiers in Nutrition.

[bb0200] Martins D., Estevinho B., Rocha F., Dourado F., Gama M. (2020). A dry and fully dispersible bacterial cellulose formulation as a stabilizer for oil-in-water emulsions. Carbohydrate Polymers.

[bb0205] Mohammed N.K., Tan C.P., Manap Y.A., Muhialdin B.J., Hussin A.S.M. (2020). Spray drying for the encapsulation of oils—A review. Molecules.

[bb0210] Molina E., Papadopoulou A., Ledward D.A. (2001). Emulsifying properties of high pressure treated soy protein isolate and 7S and 11S globulins q. Food Hydrocolloids.

[bb0215] Nahar M.K., Zakaria Z., Hashim U., Bari M.F. (2017). Effect of pH and salt concentration on protein solubility of slaughtered and non-slaughtered broiler chicken meat. Sains Malaysiana.

[bb0220] Nakonechna K., Ilko V., Berčíková M., Vietoris V., Panovská Z., Doležal M. (2024). Nutritional, utility, and sensory quality and safety of sunflower oil on the central European market. Agriculture.

[bb0225] Nicoud L., Lattuada M., Yates A., Morbidelli M. (2015). Impact of aggregate formation on the viscosity of protein solutions. Soft Matter.

[bb0230] Nikolic Z., Ðordevic V., Torbica A., Mikic A. (2012). Journal of food composition and analysis legumes seed storage proteins characterization by SDS-PAGE and lab-on-a-Chip electrophoresis. Journal of Food Composition and Analysis.

[bb0235] Peng Q., Meng Z., Luo Z., Duan H., Ramaswamy H.S., Wang C. (2023). Effect of emulsion particle size on the encapsulation behavior and oxidative stability of spray microencapsulated sweet Orange oil (Citrus aurantium var. dulcis). Foods.

[bb0240] Piñón-Balderrama C.I., Leyva-Porras C., Terán-Figueroa Y., Espinosa-Solís V., Álvarez-Salas C., Saavedra-Leos M.Z. (2020). Processes.

[bb0245] Rashidinejad A., Jafari S.M. (2020). Handbook of Food Nanotechnology.

[bb0250] Sandhya K., Leena M.M., Moses J.A., Anandharamakrishnan C. (2023). Edible oil to powder technologies: Concepts and advances. Food Bioscience.

[bb0255] Santos D., Maurício A.C., Sencadas V., Santos J.D., Fernandes M.H., Gomes P.S. (2018). Spray drying: An overview. Biomaterials-Physics and Chemistry-New Edition..

[bb0260] Schweiggert-Weisz U., Eisner P., Bader-Mittermaier S., Osen R. (2020). Food proteins from plants and fungi. Current Opinion in Food Science.

[bb0265] Sha L., Xiong Y.L. (2022). Comparative structural and emulsifying properties of ultrasound-treated pea (Pisum sativum L.) protein isolate and the legumin and vicilin fractions. Food Research International.

[bb0270] Soottitantawat A., Bigeard F., Yoshii H., Furuta T. (2005).

[bb0275] Srivastava S., Mishra H.N. (2021). Development of microencapsulated vegetable oil powder based cookies and study of its physicochemical properties and storage stability. LWT.

[bb0280] Tan L., Hong P., Yang P., Zhou C., Xiao D., Zhong T. (2019). Correlation between the water solubility and secondary structure of Tilapia-soybean protein co-precipitates. Molecules.

[bb0285] Taylor P., Benoit S.M., Afizah M.N., Ruttarattanamongkol K. (2013). Effect of pH and Temperaturae on the viscosity of texturized and commercial whey protein dispersions. International Journal of Food Properties, March.

[bb0290] Tsabet È., Fradette L. (2015). Effect of the properties of oil, particles, and water on the production of Pickering emulsions. Chemical Engineering Research and Design.

[bb0295] Tupuna-yerovi D.S., Gonz Z., Ruales J. (2021).

[bb0300] Uluko H., Liu L., Lv J.P., Zhang S.W. (2016). Functional characteristics of Milk protein concentrates and their modification. Critical Reviews in Food Science and Nutrition.

[bb0305] Ursica L., Tita D., Palici I., Tita B., Vlaia V. (2005). Particle size analysis of some water/oil/water multiple emulsions. Journal of Pharmaceutical and Biomedical Analysis.

[bb0310] Vélez-Erazo E.M., Silva I.L., Comunian T., Kurozawa L.E., Hubinger M.D. (2020). Effect of chia oil and pea protein content on stability of emulsions obtained by ultrasound and powder production by spray drying. Journal of Food Science and Technology.

[bb0315] Vignolles M.L., Lopez C., Madec M.N., Ehrhardt J.J., Méjean S., Schuck P., Jeantet R. (2009). Fat properties during homogenization, spray-drying, and storage affect the physical properties of dairy powders. Journal of Dairy Science.

[bb0320] Wang J., Xia N., Yang X., Yin S., Qi J., He X. (2012). Adsorption and dilatational rheology of heat-treated soy protein at the oil − water Interface: Relationship to structural properties. Agricultural and Food Chemistry.

[bb0325] Xiao Z., Xia J., Zhao Q., Niu Y., Zhao D. (2022). Maltodextrin as wall material for microcapsules: A review. Carbohydrate Polymers.

[bb0330] Xu X., Tao J., Wang Q., Ge J., Li J., Gao F., Gao S., Yang Q., Feng B., Gao J. (2023). A comparative study: Functional, thermal and digestive properties of cereal and leguminous proteins in ten crop varieties. LWT.

[bb0335] Yarlina V.P., Rizky A., Diva A., Zaida Z., Djali M., Andoyo R., Lani M.N. (2024). Maltodextrin concentration on the encapsulation efficiency of tempeh protein concentrate from Jack bean (*Canavalia ensiformis*): Physical, chemical, and structural properties. International Journal of Food Properties.

[bb0340] Zabot G.L., Rodrigues F.S., Ody L.P., Vin M., Herrera E., Palacin H. (2022). Encapsulation of bioactive compounds for food and agricultural applications. Polymers.

[bb0345] Zhang H., Zhao X., Chen X., Xu X. (2022). Frontiers in nutrition.

[bb0350] Zhang S., Holmes M., Ettelaie R., Sarkar A. (2020). Pea protein microgel particles as Pickering stabilisers of oil-in-water emulsions: Responsiveness to pH and ionic strength. Food Hydrocolloids.

[bb0355] Zhang W., Jin M., Wang H., Cheng S., Cao J., Kang D., Liu G. (2024). Effect of thermal treatment on gelling and emulsifying properties of soy β-conglycinin and glycinin. Foods.

[bb0360] Zhong V.W., Allen N.B., Greenland P., Carnethon M.R., Ning H., Wilkins J.T. (2021). Protein foods from animal sources, incident cardiovascular disease and all-cause mortality: A substitution analysis. International Journal of Epidemiology.

[bb0365] Zou L., Xie A., Zhu Y., McClements D.J. (2019). Cereal proteins in nanotechnology: Formulation of encapsulation and delivery systems. Current Opinion in Food Science.

