# Supplementary material for: Effect of heat treatment on physicochemical, interfacial, and encapsulation properties of pea and soy protein-based emulsions and their spray-dried powders
Source: Food Chem X. 2025 Jul 14;29:102791. doi: 10.1016/j.fochx.2025.102791 (PMC12284789; doi:10.1016/j.fochx.2025.102791)
Supplement: Supplementary file 1 — Supplementary material [file mmc1.docx]

**
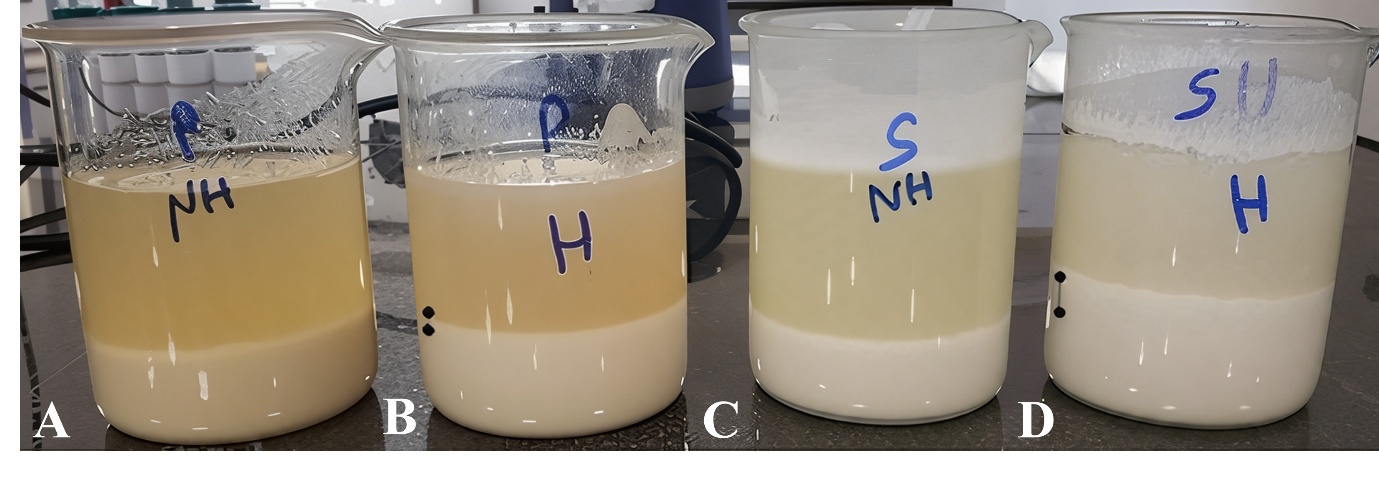
**

**Fig. S1.** Visual appearance of protein dispersion solutions: (**A**) pea protein solution (non-heated), (**B**) pea protein solution (heated), (**C**) soy protein solution (non-heated), and (**D**) soy protein suspension (heated).


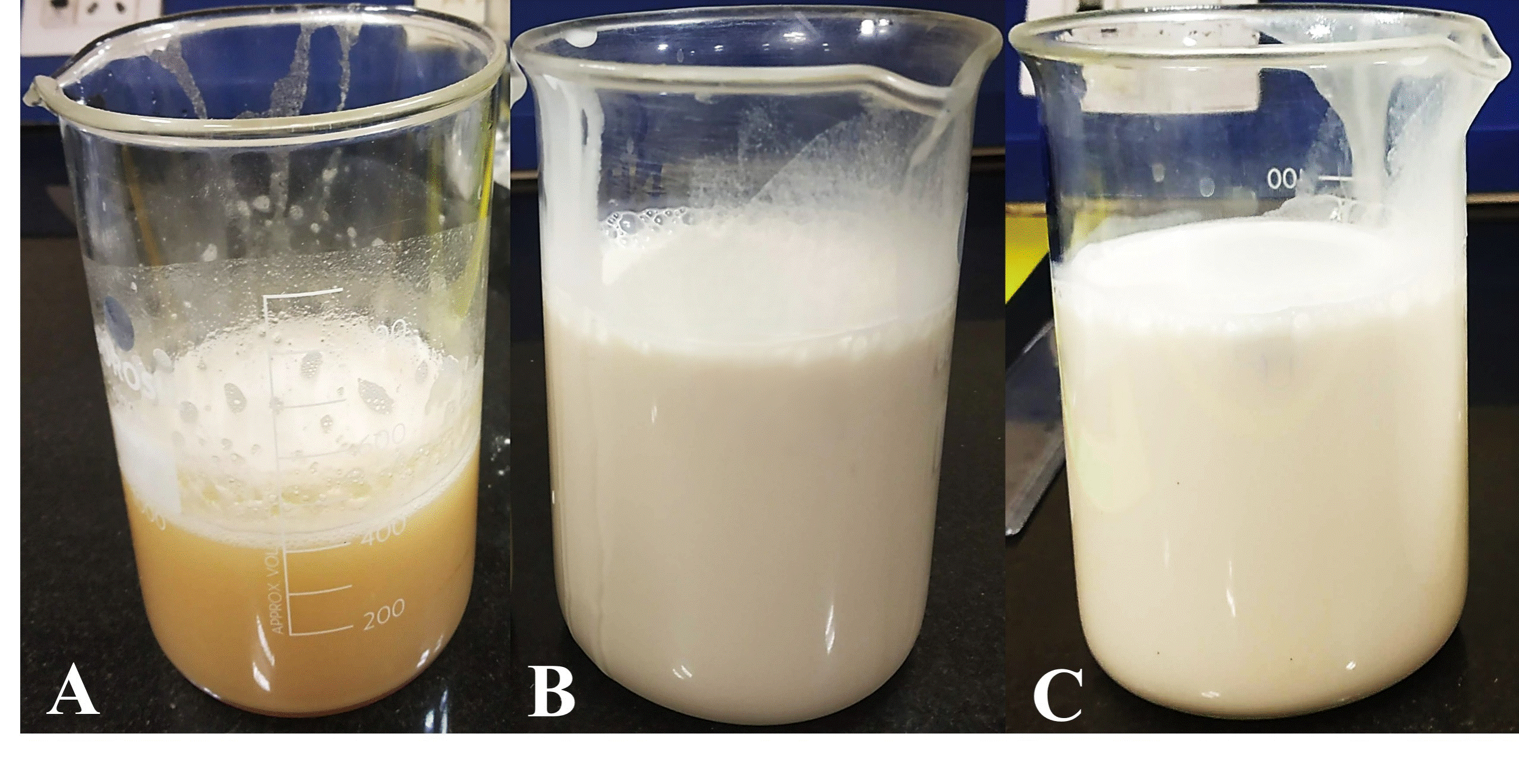


**Fig. S2.** Visual appearance of pea protein emulsions: (**A**) pea protein emulsion before homogenization, (**B**) P65-mildly heated pea protein emulsion after homogenization, and (**C**) P85-moderately heated pea protein emulsion after homogenization.


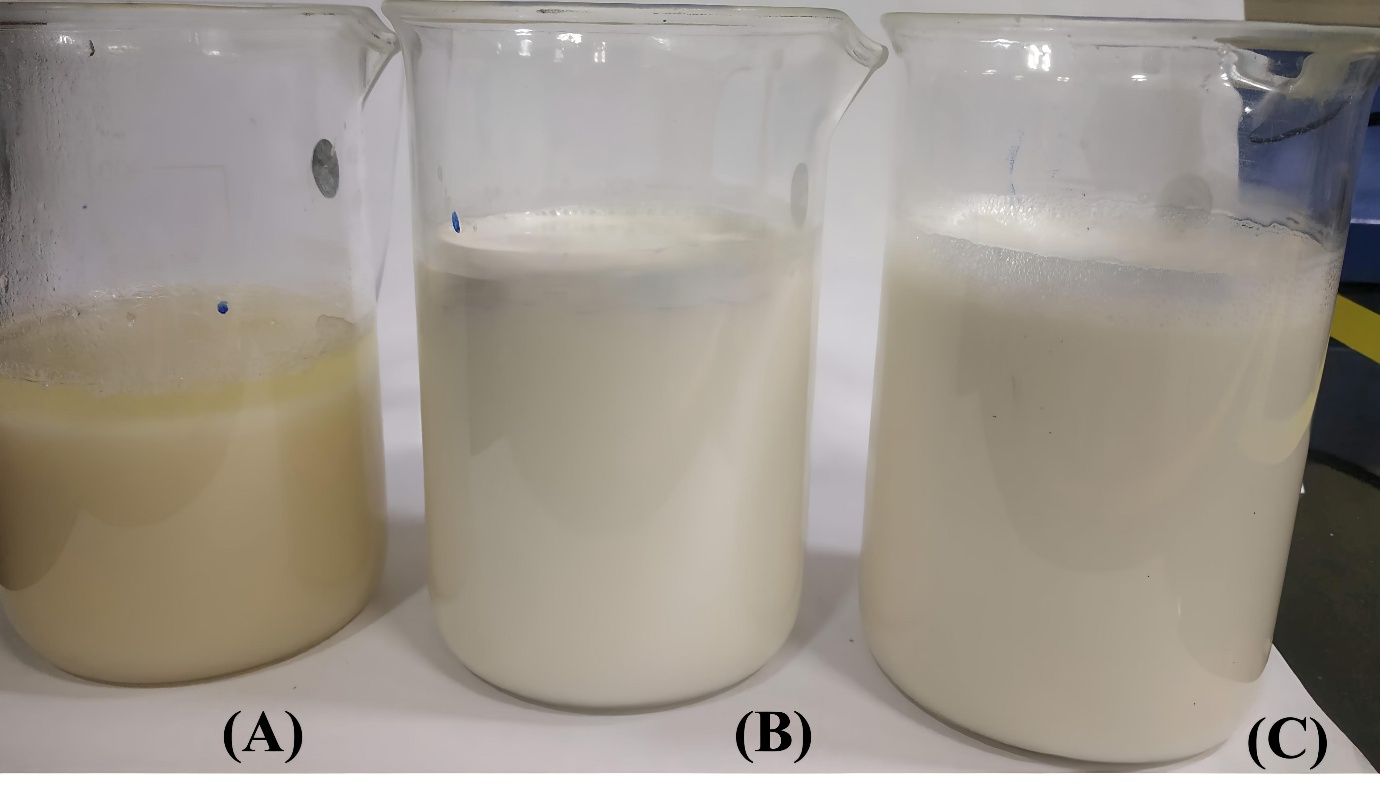


**Fig. S3.** Illustration of soy protein emulsions: (**A**) soy protein emulsion before homogenization, **(B**) S65-mildly heated soy protein emulsion after homogenization, and (**C**) S85-moderately heated soy protein emulsion after homogenization.


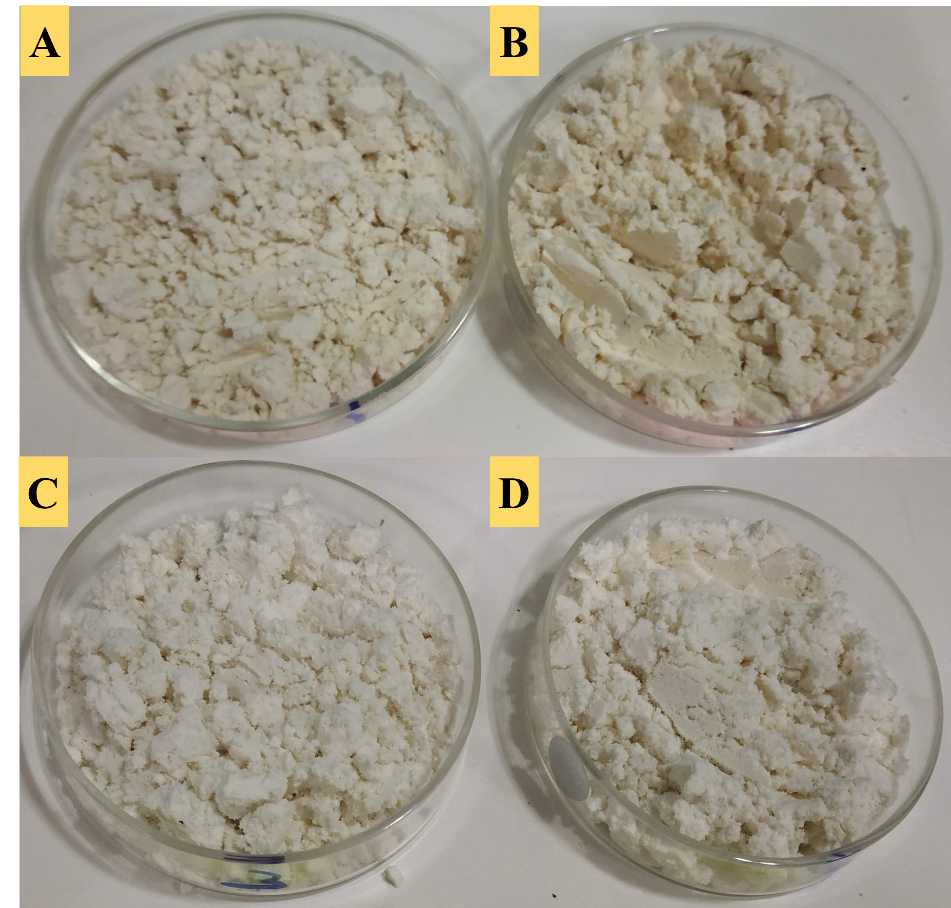


**Fig. S4.** Spray-dried emulsion powders: (**A**) mildly heated pea protein, (**B**) moderately heated pea protein, (**C**) mildly heated soy protein, and (**D**) moderately heated soy protein.


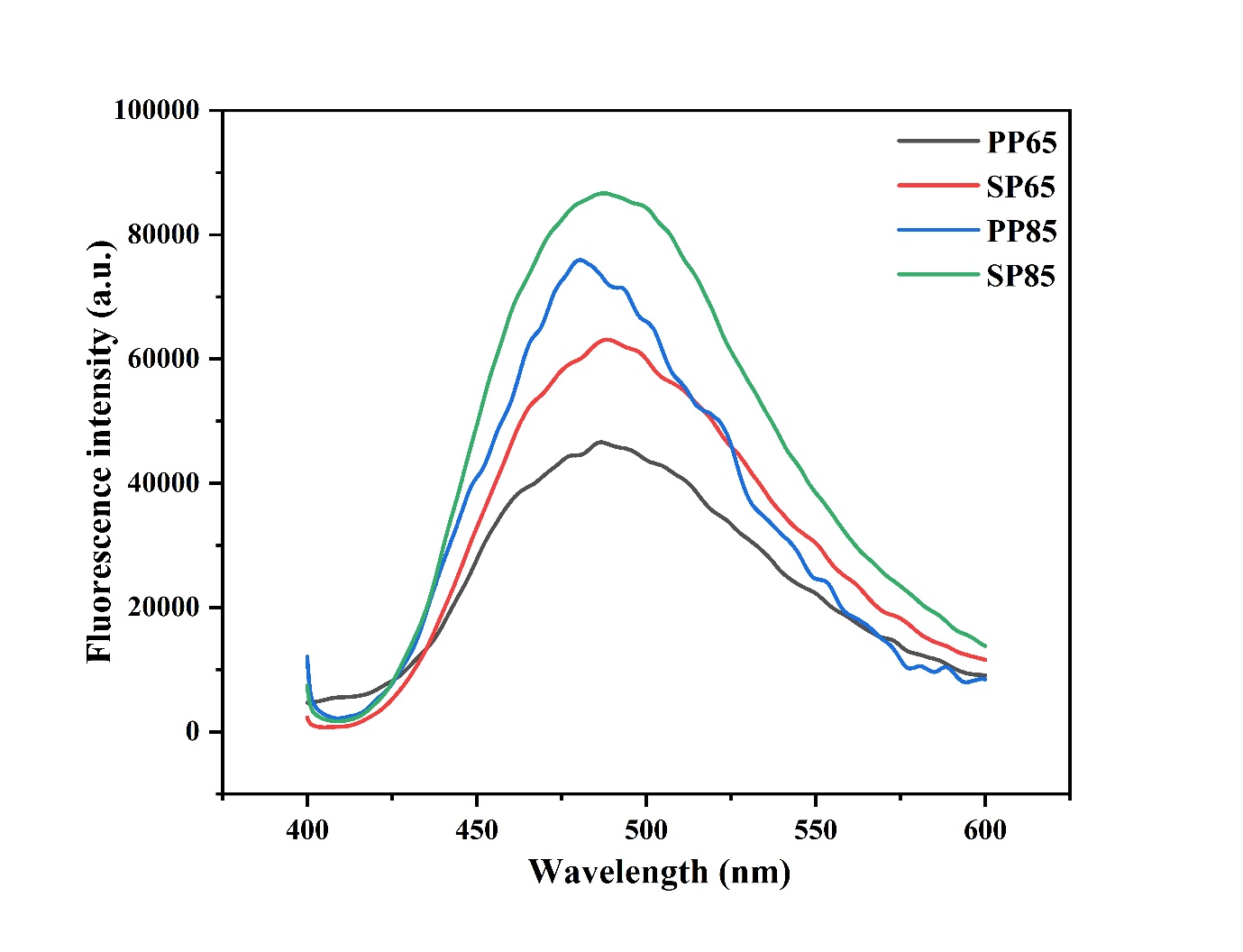


**Fig. S5.** Effect of heat treatment on surface hydrophobicity of protein (Analyzed by ANS fluorescence assay method).


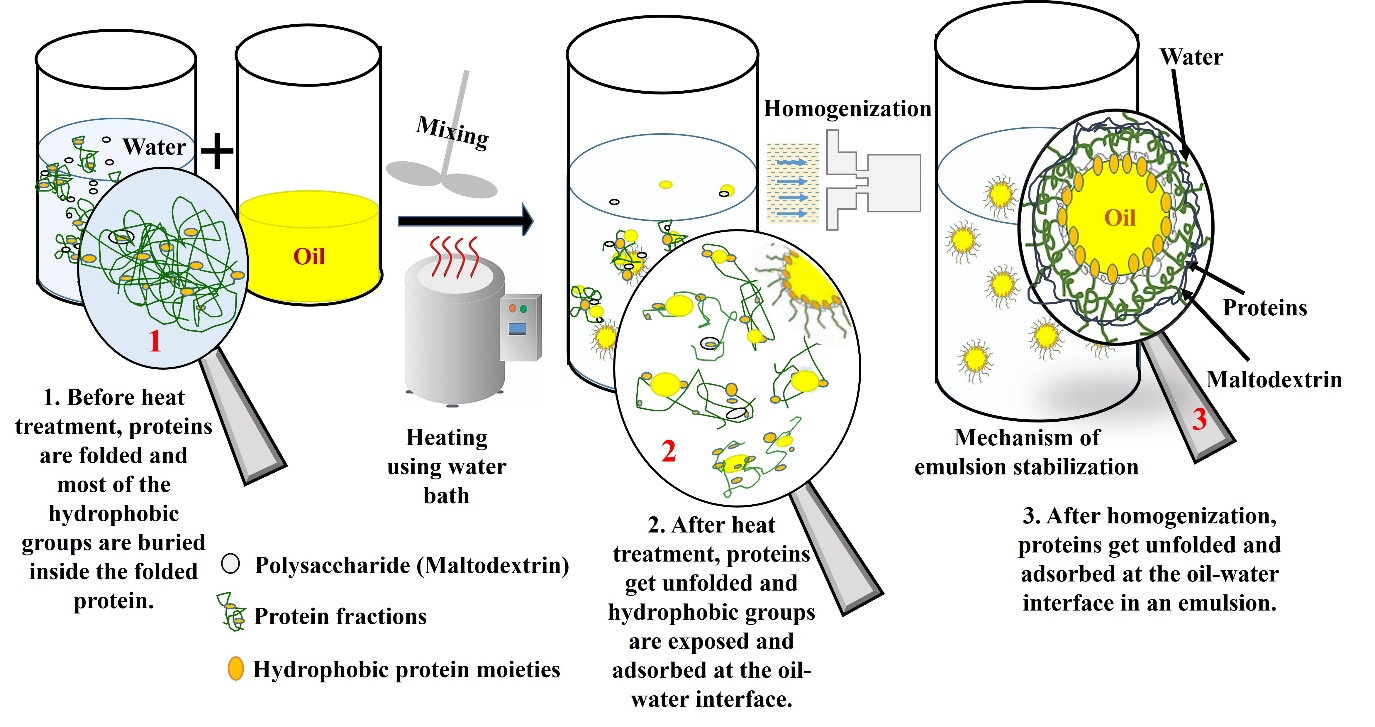


**Fig. S6.** Effect of heat treatment on protein adsorption at the oil-water interface.


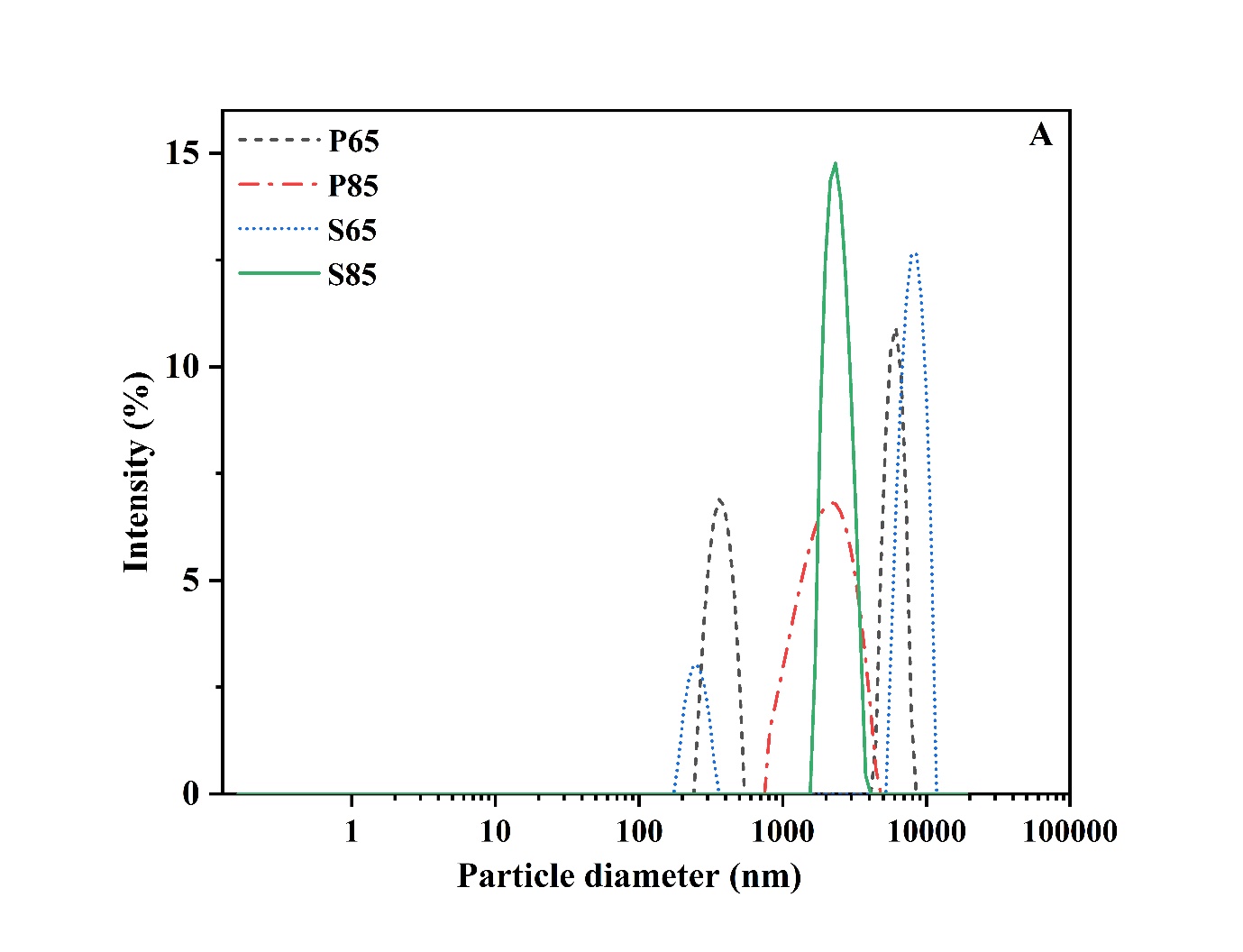


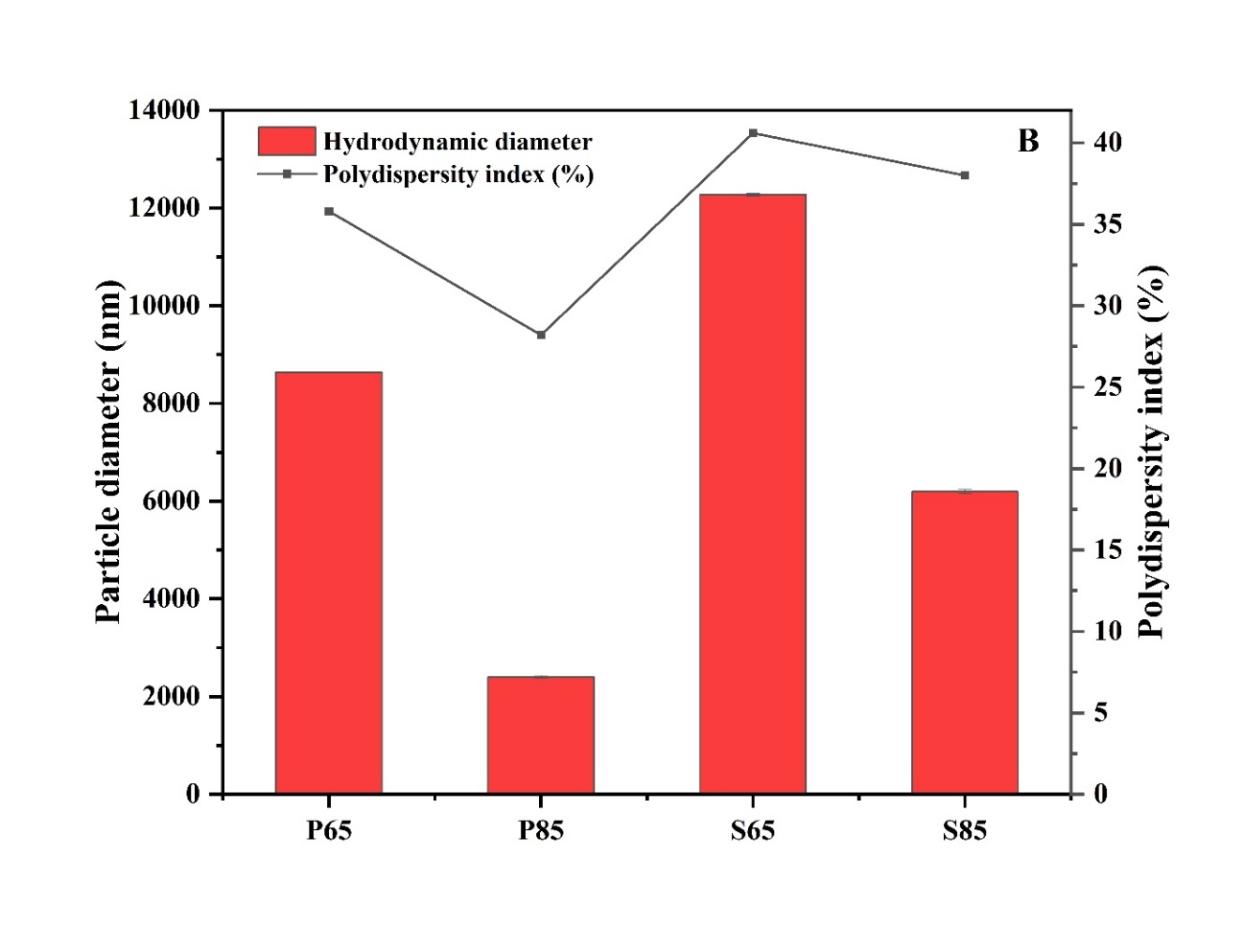


**Fig. S7.** (**A**) Particle size distribution of emulsions. (**B**) Particle diameter and polydispersity index (PDI) of emulsions.
